# Associations of work-related extended availability with overweight and obesity among Chinese workers

**DOI:** 10.3389/fpubh.2026.1907128

**Published:** 2026-09-03

**Authors:** Huiying Wang, Xin Li

**Affiliations:** 1 School of Labor Economics, Capital University of Economics and Business, Beijing, China; 2 School of Marxism, Hebei University of Engineering, Handan, China

**Keywords:** body mass index, obesity, occupational health, overweight, work-related extended availability

## Abstract

**Objective:**

Work-related extended availability (WREA) has become an increasingly salient feature of the contemporary labor market and may be associated with workers' health outcomes. Nevertheless, the associations of WREA with overweight and obesity remain insufficiently explored. This study aimed to examine the associations of WREA with overweight and obesity among Chinese workers.

**Methods:**

Using data from the 2020 and 2022 waves of the China Family Panel Studies, we constructed an analytical sample of 16,969 observations and employed ordered logistic regression models (ologit) to examine the associations of WREA with overweight and obesity. The models adjusted for a comprehensive set of covariates, including demographic characteristics, health status and behaviors, job characteristics, and socioeconomic factors. A series of sensitivity analyses was performed to assess the consistency of the main findings. We also conducted exploratory analyses to examine heterogeneity across demographic subgroups, potential effect modification by work- and family-related factors, variation across study-defined WREA categories, and the longitudinal association of WREA with subsequent overweight and obesity.

**Results:**

The reported prevalence rates of overweight and obesity were 31.95 and 9.92%, respectively, and 53.29% of workers were exposed to WREA. Overall, WREA was positively associated with the prevalence of overweight (average marginal effect = 0.019, 95% CI = 0.011 to 0.028) and obesity (average marginal effect = 0.012, 95% CI = 0.007 to 0.019). These associations remained broadly consistent across a series of sensitivity analyses. In exploratory heterogeneity analyses, the estimated associations tended to be more pronounced among men, individuals with medium educational attainment, and those aged 30–50 years. Additionally, the estimated associations also appeared to vary by employment status and the financially support for parents. Exploratory category-specific analyses suggested variation across the study-defined WREA categories, with estimates tending to be larger for contract-based and digitally enabled WREA. However, an exploratory longitudinal analysis did not provide sufficient evidence of associations between WREA and subsequent overweight or obesity.

**Conclusions:**

WREA was positively associated with the prevalence of overweight and obesity. As WREA becomes an increasingly salient feature in modern workplaces, its potential occupational health implications warrant further investigation and attention.

## 1 Introduction

The prevalence of overweight and obesity has increased sharply in recent years. Existing epidemiological research has shown that the combined prevalence of overweight and obesity among Chinese adults rose from 30.1% in 2004 to 56.9% in 2023, nearly doubling over this period. In the absence of effective interventions, this rate is projected to rise further to 65.5% by 2030 and 72.2% by 2035 (1). Overweight and obesity not only increase the risk of a wide range of chronic diseases, but also incur substantial health care costs and impose a considerable burden on society and the economy (2, 3). Currently, the obesity epidemic has become a major public health challenge in China. Meanwhile, researchers have conducted extensive research on the drivers of overweight and obesity across multiple dimensions. Beyond individual genetic and behavioral factors, a growing body of research has increasingly highlighted the important role of obesogenic environments in the pathogenesis of overweight and obesity, including work arrangements, residential environments, and prevailing social norms (4, 5). Notably, given that China's labor force accounts for more than half of the total population, occupational exposures deserve particular attention as potential environmental determinants of overweight and obesity.

With the widespread use of digital office platforms and instant communication tools, work availability beyond regular working hours has become an increasingly salient feature of contemporary workplaces (6). Work-related extended availability (WREA) refers to a condition in which workers remain reachable for work-related matters, including during non-work hours (7, 8). In practice, workers primarily maintain such availability by keeping their communication devices switched on throughout the day. Unlike traditional on-call work, which usually requires employees to remain available during predefined duty periods, WREA is generally not regulated by formal agreements. Enabled by information and communication technologies, it extends work availability beyond regular working hours, irrespective of physical location, and operates as an informal normative practice rather than a formally prescribed duty (9). This increases workers' reachability and heightens the uncertainty of work interruptions during private time. By blurring the boundary between work and private life, WREA may reshape workers' daily routines, recovery opportunities, and health-related behaviors, making it an important occupational exposure in modern work environments (10).

Existing research has shown that WREA may be negatively associated with recovery experiences, psychological detachment, and sleep quality, while being positively associated with stress, negative affect, and emotional exhaustion (8, 11–13). Given the links between these factors and obesity-relevant behaviors and physiological processes (14–16), WREA may also be associated with overweight or obesity. However, the relationships between WREA and overweight and obesity, remain underexplored in the literature. Furthermore, few studies on the association of WREA and health outcomes have examined heterogeneity across population groups and under different work and family circumstances.

This study aimed to systematically examine the associations of WREA with overweight and obesity. We also assessed potential heterogeneity in these associations across population subgroups. To inform more targeted recommendations, we further explored potential effect modification by employment status, access to rest days, child-rearing responsibilities, and the support for parents, thereby considering factors from both work and family contexts. Given the potential heterogeneity of WREA, we also conducted exploratory analyses using operational classifications based on labor contract status and underlying drivers to examine possible variation in the association with overweight and obesity across WREA categories. Finally, we conducted an exploratory longitudinal analysis to examine the associations of WREA with subsequent overweight and obesity. As WREA is likely to remain a common feature in the coming years, clarifying its association with overweight and obesity can provide theoretical and empirical support for occupational health management.

## 2 Methods

### 2.1 Sample selection

This study uses data from the China Family Panel Studies (CFPS), a national, comprehensive social follow-up survey project. In 2010, the CFPS conducted its baseline survey across 25 provinces, municipalities, and autonomous regions in China. Since then, follow-up surveys have been carried out every two 2 years. To date, survey data from 2010 to 2022 have been made available to the academic community and the public. The CFPS collects information at the individual, household, and community levels, reflecting changes in the Chinese society, economy, population, education, and health. With its rich set of variables and large sample size, the CFPS is well suited to this study.

**Figure 1:**
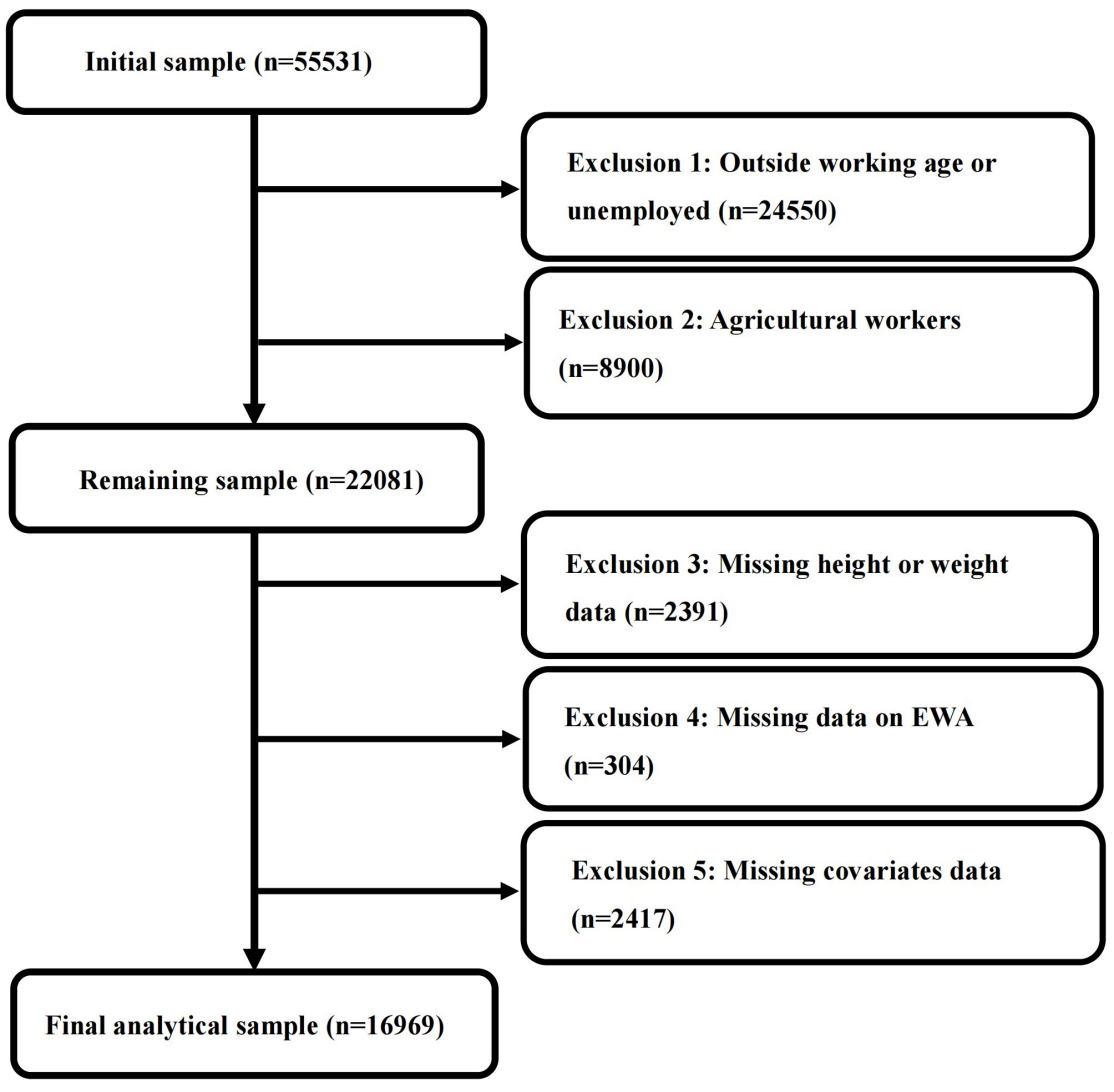
Flowchart of sample selection.

Since the survey items related to the core independent variable were first introduced in 2020, this study ultimately used data from the 2020 and 2022 survey waves, comprising an initial sample of 55,531 observations. During this period, digital tools were widely adopted, while WREA became increasingly prevalent across China. Given the study's focus on occupational health, we first excluded 24,550 individuals who were either outside the working-age range or unemployed. We then excluded 8,900 agricultural workers. The reason is that agricultural work is more strongly shaped by natural factors such as weather, seasonality, and crop cycles. WREA, which is mainly driven by information technology and organizational expectations, is less applicable to agricultural workers in this study. We sequentially excluded 2,391 observations with missing height or weight data, 304 observations with missing WREA data, and 2,417 observations with missing covariate data. In the end, the final analytical sample therefore consisted of 16,969 observations with complete data on all variables used in the analyses. The sample selection process is shown in Figure 1.

### 2.2 Measures

#### 2.2.1 Dependent variable: overweight and obesity status

Overweight and obesity status served as the dependent variable in this study. Consistent with international practice, we used body mass index (BMI) as the measure of overweight and obesity. Height values outside 1.2–2.1 m and weight values outside 22–317 kg were treated as biologically implausible and excluded from analysis (17). Then we calculated BMI as the ratio of self-reported weight in kilograms to the square of self-reported height in meters.

According to the *Guidelines for Weight Management (2024 Edition)* issued by the National Health Commission of China, participants were classified as underweight if their BMI was below 18.5 kg/m^2^ (coded as 0), normal weight if their BMI was 18.5 to < 24.0 kg/m^2^ (coded as 1), overweight if their BMI was 24.0 to < 28.0 kg/m^2^ (coded as 2), and obesity if their BMI was 28.0 kg/m^2^ or higher (coded as 3). In the robustness analysis, we use BMI as a continuous quantitative measure of overweight and obesity status and adopt the WHO classification standards for Asians to conduct sensitivity analysis.

#### 2.2.2 . Independent variable: work-related extended availability

Work-related extended availability (WREA) was the primary exposure variable in this study. Based on the questionnaire item “Do you need to keep your mobile phone switched on 24 h a day and remain on call for job demands?” Respondents who answered “Yes” were classified as being exposed to WREA and assigned a value of 1. In contrast, those who answered “No” were assigned a value of 0.

#### 2.2.3 . Covariate variable

Drawing on existing literature, control variables include demographic characteristics, health status and behaviors, job characteristics, and socioeconomic factors (18, 19). Demographic variables included age, age squared, gender, years of education, and marital status. Health status and health behavior variables covered smoking and drinking habits, protein and vegetable intake, and chronic disease history. Considering that other job characteristics may also affect overweight and obesity, we further controlled night shift work and Working hours. Additionally, we controlled for a range of socioeconomic factors, including per capita household income, urban–rural status, personal economic position, and social position. We also incorporated fixed effects for occupation, year, and province to account for unobserved heterogeneity at these levels. For the continuous control variables, such as per capita household income and working hours, we winsorized values at the 1st and 99th percentiles to reduce the influence of extreme values.

### 2.3 Statistical analysis

We conducted descriptive analyses for the overall sample, workers exposed to WREA, and those not exposed to WREA. Continuous variables were presented as means and standard deviations, whereas categorical variables were presented as frequencies and percentages. Between-group differences were assessed using *t*-tests for continuous variables and chi-square tests for categorical variables.

An ordered logistic regression model was used to examine the associations of WREA with overweight and obesity. This study used the oparallel command in STATA to test for proportional-odds assumption. Since *P* >0.05(*P* = 0.071, χ^2^ = 46.782, df = 34), it was concluded that the proportional-odds assumption was not rejected. We further employed instrumental variable methods, propensity score matching, and a series of robustness checks to evaluate the sensitivity of the observed associations to potential endogeneity, selection bias, and alternative analytical specifications. To explore potential heterogeneity, we subsequently conducted subgroup analyses by gender, educational attainment, and age, together with formal tests of between-group differences. Complementing the subgroup analyses, we performed exploratory interaction analyses by incorporating interaction terms into the baseline regression models to examine whether the associations varied by work and family factors. We also conducted exploratory category-specific analyses by classifying WREA into categories and including the corresponding binary indicators in the baseline regression models to assess whether associations with overweight and obesity differed across WREA forms. Finally, we explored the associations of WREA with subsequent overweight and obesity using longitudinal data.

Because the CFPS is a longitudinal survey, some respondents appeared in both the 2020 and 2022 waves. To account for within-individual correlation arising from repeated observations across survey waves, all standard errors were clustered at the individual level. All statistical analyses were performed using Stata version 17. All statistical tests were two-tailed. The significance level was set at *p* < 0.05.

## 3 Results

### 3.1 Descriptive analysis

Table 1 presents the sociodemographic characteristics of the study sample. Overall, 53.29% of workers were exposed to WREA and required to remain on call 24 h a day. The prevalence of overweight and obesity was 31.95 and 9.92%, respectively. These results are consistent with prior Chinese studies (20). Workers exposed to WREA had significantly higher overweight rates, obesity rates, and BMI values than unexposed workers (*P* < 0.001). Further comparisons indicated that workers exposed to WREA were more likely to be male, older, less educated, and to have a spouse. They also reported higher rates of smoking and alcohol consumption. This group also had lower protein intake, a higher risk of chronic disease, a greater likelihood of working night shifts, longer daily work hours, lower household per capita income, higher self-rated social status and personal income status. No statistically significant differences were observed between the two groups in fruit and vegetable intake, and residence status.

**Table 1:** Descriptive statistics of the study sample (*n* = 16,969).

Variables	Definition	Total	WREA		*P*-value
			Yes	No	
Overall		16,969 (100%)	9,043 (53.29%)	7,926 (46.71%)	
Obesity status	0 = underweight (BMI < 18.5)	958 (5.65%)	424 (4.69%)	534 (6.74%)	< 0.001
	1 = normal (18.5 < BMI ≤ 24)	8,906 (52.48%)	4,500 (49.76%)	4,406 (55.59%)	
	2 = overweight (24 < BMI ≤ 28)	5,422 (31.95%)	3,074 (33.99%)	2,348 (29.62%)	
	3 = obesity (BMI > 28)	1,683 (9.92%)	1,045 (11.56%)	638 (8.05%)	
BMI	kg/m^2^	23.493 (3.451)	23.815 (3.493)	23.124 (3.3640)	< 0.001
Age	Age of the individual	38.951 (10.989)	40.252 (10.964)	37.467 (10.8288)	< 0.001
Age2	Square of an individual's age	1637.996 (899.966)	1740.473 (913.956)	1,521.078 (869.143)	< 0.001
Gender	1 = Male	9,946 (58.61%)	5,885 (65.08%)	4,061 (51.24%)	< 0.001
	0 = female	7,023 (41.39%)	3,158 (34.92%)	3,865 (48.76%)	
Education	Years of education	11.169 (3.976)	11.089 (3.948)	11.261 (4.006)	0.004
Spouse	1 = have a spouse (married or cohabitating)	13,341 (78.62%)	7,368 (81.48%)	5,973 (75.36%)	< 0.001
	0 = don't have a spouse (unmarried, divorced, or widowed)	3,628 (21.38%)	1,675 (18.52%)	1,953 (24.64%)	
Smoker	1 = yes	5,550 (32.71%)	3,432 (37.95%)	2,118 (26.72%)	< 0.001
	0 = no	11,419 (67.29%)	5,611 (62.05%)	5,808 (73.28%)	
Drinker	1 = yes	2,511 (14.80%)	1,615 (17.86%)	896 (11.30%)	< 0.001
	0 = no	14,458 (85.20%)	7,428 (82.14%)	7,030 (88.70%)	
Protein intake	1 = consumed protein-containing food in the past week	16,033 (94.48%)	8,502 (94.02%)	7,531 (95.02%)	0.004
	0 = did not consume protein-containing food	936 (5.52%)	541 (5.98%)	395 (4.98%)	
Vegetable intake	1 = consumed fruit or vegetables in the past week	16,767 (98.81%)	8,936 (98.82%)	7,831 (98.80%)	0.927
	0 = did not consume fruit or vegetable	202 (1.19%)	107 (1.18%)	95 (1.20%)	
Chronic diseases	1 = with chronic disease	1,584 (9.33%)	927 (10.25%)	657 (8.29%)	< 0.001
	0 = without chronic disease	15,385 (90.67%)	8,116 (89.75%)	7,269 (91.71%)	
Night-shift	1 = required to work night shifts	1,102 (6.49%)	768 (8.49%)	334 (4.21%)	< 0.001
	0 = not required	15,867 (93.51%)	8,275 (91.51%)	7,592 (95.79%)	
Working hours	Ordinary weekly working hours, including paid and unpaid overtime over the past 12 months.	52.837 (19.199)	54.1614 (20.351)	51.3274 (17.675)	< 0.001
Income	Log of per capita household income	10.297 (0.845)	10.279 (0.841)	10.317 (0.850)	0.003
Income_Status	Self-rated local income status, 1–5	2.854 (0.909)	2.888 (0.932)	2.815 (0.881)	< 0.001
Social_ Status	Self-rated local social status, 1–5	2.859 (0.951)	2.919 (0.977)	2.790 (0.915)	< 0.001
Residence	1 = urban	11,023 (64.96%)	5,865 (64.86%)	5,158 (65.08%)	0.764
	0 = rural	5,946 (35.04%)	3,178 (35.14%)	2,768 (34.92%)	

Chi-square test and *t*-test were applied to detect distributed differences for categorical and continuous variables.

### 3.2 Associations of WREA with overweight and obesity

We employed an ordered logistic regression model to examine the association of WREA with overweight and obesity, and we reported average marginal effects. As shown in Table 2, after adjustment for covariates, WREA was positively associated with higher BMI categories in the ordered logit model (β = 0.149, 95% CI: 0.083 to 0.216), with the 95% confidence interval excluding zero. Marginal effect estimates indicated that higher WREA was associated with a 1.9 percentage-point increase in the probability of overweight (95% CI: 0.011 to 0.028) and a 1.2 percentage-point increase in the probability of obesity (95% CI: 0.007 to 0.019). Correspondingly, the probabilities of underweight and normal weight decreased by 0.7 percentage points (95% CI: −0.011 to −0.004) and 2.4 percentage points (95% CI: −0.036 to −0.014), respectively.

**Table 2:** Associations between WREA and overweight and obesity in the main analysis.

Variable	Coefficient	Marginal effects			
		Underweight	Normal weight	Overweight	Obesity
WREA	0.149^***^ [0.083,0.216]	−0.007^***^ [−0.011, −0.004]	−0.024^***^ [−0.036, −0.014]	0.019^***^ [0.011, 0.028]	0.012^***^ [0.007, 0.019]
Age	0.172^***^ [0.145,0.198]	−0.008^***^ [−0.010, −0.007]	−0.028^***^ [−0.033, −0.024]	0.022^***^ [0.019, 0.026]	0.015^***^ [0.012, 0.017]
Age 2	−0.001^***^ [−0.002, −0.001]	0.000^***^ [0.000, 0.000]	0.0003^***^ [0.000, 0.000]	−0.0002^***^ [0.000, −0.000]	−0.0001^***^ [0.000, −0.000]
Gender	0.955^***^ [0.864, 1.046]	−0.049^***^ [−0.055, −0.043]	−0.158^***^ [−0.172, −0.144]	0.125^***^ [0.114, 0.137]	0.082^***^ [0.073, 0.090]
Education	−0.032^***^ [−0.043, −0.021]	0.002^***^ [0.001, 0.002]	0.005^***^ [0.004, 0.007]	−0.0043^***^ [−0.006,−0.003]	−0.003^***^ [−0.004, −0.002]
Spouse	0.424^***^ [0.317,0.531]	−0.022^***^ [−0.027, −0.016]	−0.070^***^ [−0.088, −0.052]	0.056^***^ [0.042, 0.070]	0.036^***^ [0.027, 0.046]
Smoker	−0.131^***^ [−0.220, −0.042]	0.007^***^ [0.002, 0.011]	0.022^***^ [0.007, 0.036]	−0.017^***^ [−0.029, −0.006]	−0.011^***^ [−0.019,−0.004]
Drinker	−0.042 [−0.139, 0.054]	0.002 [−0.003, 0.007]	0.007 [−0.009, 0.023]	−0.006 [−0.018, 0.007]	−0.004 [−0.012, 0.005]
Protein intake	0.124^*^ [−0.017, 0.266]	−0.006^*^ [−0.014, 0.001]	−0.021^*^ [−0.044, 0.003]	0.016^*^ [−0.002, 0.035]	0.011^*^ [−0.002, 0.023]
Vegetable intake	0.315^**^ [0.022, 0.607]	−0.016^**^ [−0.031, −0.001]	−0.052^**^ [−0.100, −0.004]	0.041^**^ [0.003, 0.080]	0.027^**^ [0.002, 0.052]
Chronic diseases	0.228^***^ [0.117, 0.339]	−0.012^***^ [−0.017, −0.006]	−0.038^***^ [−0.056, −0.019]	0.030^***^ [0.015, 0.044]	0.020^***^ [0.010, 0.029]
Night-shift	0.168^**^ [0.035, 0.300]	−0.009^**^ [−0.015, −0.002]	−0.028^**^ [−0.050, −0.006]	0.022^**^ [0.005, 0.039]	0.014^**^ [0.003, 0.026]
Working hours	0.000 [−0.001, 0.002]	−0.000 [−0.000, 0.000]	−0.000 [−0.000, 0.000]	0.000 [−0.000, 0.000]	0.000 [−0.000, 0.000]
Income	0.034 [−0.011, 0.080]	−0.002 [−0.004, 0.001]	−0.006 [−0.013, 0.002]	0.004 [−0.002, 0.011]	0.003 [−0.001, 0.007]
Income_Status	0.025 [−0.010, 0.061]	−0.001 [−0.003, 0.001]	−0.004 [−0.010, 0.002]	0.003 [−0.001, 0.008]	0.002 [−0.001, 0.005]
Social_ Status	0.005 [−0.013, 0.023]	−0.000 [−0.001, 0.001]	−0.001 [−0.004, 0.002]	0.001 [−0.002, 0.003]	0.000 [−0.001, 0.002]
Residence	0.126^***^ [0.047, 0.204]	−0.006^***^ [−0.011, −0.002]	−0.021^***^ [−0.034, −0.008]	0.017^***^ [0.006, 0.027]	0.011^***^ [0.004, 0.017]
Occupation FE	YES				
Year FE	YES				
Province FE	YES				
N	16,969				
Pseudo R2	0.0618				

95% confidence intervals are shown in brackets. ^*^, ^**^, and ^***^ indicate significance at the 10%, 5%, and 1% levels, respectively. Clustering was performed at the individual level. Unless otherwise specified, this footnote applies to all subsequent tables as well.

Among the covariates, age showed a significant inverted U-shaped association with both overweight and obesity. Workers who were male, had fewer years of education, were married, non-smokers, consumed protein and fruits/vegetables, suffered from chronic diseases, worked night shifts daily, and lived in urban areas were more likely to be overweight or obese. Alcohol consumption, working hours, self-rated personal income status, self-rated social status, and per capita household income were not significantly associated with overweight and obesity, respectively.

### 3.3 Sensitivity analyses

To examine whether the observed association was sensitive to potential reverse causality or omitted-variable bias, we conducted an instrumental variable analysis as a supportive sensitivity analysis. Following a leave-one-out approach, we constructed the instrumental variable (*ave_WREA*) as the mean WREA exposure among other workers within the same province–occupation–survey-wave cell. Across 2,044 province–occupation–survey-wave cells, cell sizes ranged from 1 to 157 participants, with a mean of 8.30. Because leave-one -out means may be unstable in very small cells, cells containing fewer than five participants were excluded from the IV analysis. A valid instrument must satisfy the relevance condition and the exclusion restriction. First, this instrument variable satisfies the relevance condition as individual work schedules are generally influenced by the average employment practices of peers in the same province and occupation. As shown in columns (1, 3, 5, 7 and 9) of Table 3, the first-stage Wald χ^2^ statistics are highly significant (*p* < 0.001), and the Kleibergen-Paap rk Wald F statistics exceed the critical threshold of 10. These results provided strong empirical support for instrument relevance and minimizing concerns regarding weak instruments. Second, to strengthen the plausibility of the exclusion restriction, we control for potential confounding channels by incorporating provincial-level variables that capture regional economic development and lifestyle changes, such as the log of GDP per capita, the urbanization rate, and the industrial structure. We also adjusted for individuals' subjective perceptions of employment-related issues as a proxy for labor market pressure and job-related stress. By mitigating these potential confounding channels, the inclusion of these covariates enhances the plausibility of the exclusion restriction.

**Table 3:** Sensitivity analyses: instrumental variable approach.

Variable	Conditional mixed process				Two-stage residual inclusion(2SRI)				Two-stage least squares(2SLS)	
	Cluster at the individual level		Cluster at the province–occupation–survey wave level		cluster at the individual level		Cluster at the province–occupation–survey wave level		Cluster at both individual and province–occupation–survey wave level	
	First-stage	Second-stage	First-stage	Second-stage	First-stage	Second-stage	First-stage	Second-stage	First-stage	Second-stage
	(1)	(2)	(3)	(4)	(5)	(6)	(7)	(8)	(9)	(10)
WREA		0.417^**^ [0.060, 0.774]		0.417^**^ [0.068, 0.766]		0.572^**^ [0.016, 1.134]		0.572^**^ [0.036, 1.131]		0.357^***^ [0.115, 0.598]
Ave_WREA	1.008^***^ [0.848, 1.168]		1.008^***^ [0.832, 1.184]		1.632^***^ [1.369, 1.895]		1.632^***^ [1.340, 1.923]		0.371^***^ [0.305, 0.435]	
Atanhrho_12	−0.206^*^ [−0.438, 0.025]		−0.206^*^ [−0.435, 0.023]							
Residual						−0.211^*^ [−0.431, 0.010]		−0.211^*^ [−0.427, 0.006]		
Wald χ^2^	153.130 (*P* < 0.001)		126.090 (*P* < 0.001)		147.780 (*P* < 0.001)		120.620 (*P* < 0.001)			
KP rk Wald F									123.947	
Occupation FE	YES	YES	YES	YES	YES	YES	YES	YES	YES	YES
Year FE	YES	YES	YES	YES	YES	YES	YES	YES	YES	YES
Province FE	YES	YES	YES	YES	YES	YES	YES	YES	YES	YES
N	9,805	9,805	9,805	9,805	9,805	9,805	9,805	9,805	9,805	9,805

Due to space constraints, only coefficient values are reported in the table. The first stage of 2SRI employed a logit regression, while the first stage of CMP employed a probit regression. ^*^, ^**^, and ^***^ indicate significance at the 10%, 5%, and 1% levels, respectively.

We applied the conditional mixed process (CMP), two-stage residual inclusion (2SRI) and two-stage least squares (2SLS) methods to assess the sensitivity of the observed association to potential endogeneity. Since the CMP command in Stata 17 cannot run the ordered logit model, we used the ordered probit model for the second-stage regression within the CMP framework. Additionally, two-way clustering at the individual and province–occupation–survey-wave levels was implemented in 2SLS. For CMP and 2SRI, standard errors are clustered separately at each level because joint two-way clustering is computationally infeasible. The 95% confidence intervals for both the atanhrho_12 parameter and the 2SRI residual contained zero, indicating insufficient evidence of endogeneity. Nevertheless, these results were retained for sensitivity analyses. Columns (1) and (3) of Table 3 present the first-stage regression results. The coefficients of the instrumental variable were significantly positive, indicating that the relevance condition was satisfied. Columns (2) and (4) of Table 3 report the second-stage results. The coefficients of WREA remained significantly positive and consistent with the baseline regression results under both clustering specifications. The 2SRI results in columns (5) to (8) and the 2SLS results in columns (9) and (10) were also broadly consistent with the main findings, further supporting the sensitivity of our main conclusions.

The baseline estimates may also be affected by selection bias, as workers may self-select into jobs requiring WREA. We conducted sensitivity analyses using three propensity score matching methods: one-to-one nearest-neighbor matching with replacement, radius matching with a caliper of 0.03, and kernel matching with a bandwidth of 0.06. We treated individuals exposed to WREA as the treatment group and estimated the average treatment effect on the treated (ATT). Then we re-estimated the models in the matched samples using the corresponding matching weights. When a control observation was matched to multiple treated observations, its repeated use was accounted for through the matching weight. The standard errors in the post-matching regressions were clustered at the individual level. Prior to reporting the matched estimates, we performed both a common support test and a balance test to assess the quality of the matching. Table 4 reports the results of the common support test. Only seven treated observations fall outside the common support region, while the remaining 16,962 observations lie within it. Table 5 presents the overall balance test results. Compared with the pre-matching sample, the post-matching sample shows substantial reductions in the pseudoR^2^, LR χ^2^, and standardized mean differences. Overall, these results indicate that the matching procedure performs well and substantially improves covariate balance. Due to space constraints, the covariate balance test results and Love plots for all three matching methods are reported in Supplementary Tables 1–3 and Supplementary Figures 1–3, respectively. After 1:1 nearest-neighbor, radius, and kernel matching, the absolute standardized mean differences (SMD) for all covariates fell below the 10% threshold, with the maximum SMD dropping from 28.3 to 2.2%, 2.2, and 2.5%, and the mean SMD from 12.0 to 0.9%, 0.8, and 1.1%, respectively. As shown in Table 6, the average treatment effect on the treated (ATT) was significantly positive across all three matching specifications. Meanwhile, the direction and statistical significance of the WREA coefficient were consistent with those observed in the baseline model. Taken together, these findings suggest that the positive associations of WREA with overweight and obesity are robust to propensity score matching.

**Table 4:** Sample distribution within the common support region.

Treatment status	Off support	On support	Total
Untreated	0	7,926	7,926
Treated	7	9,036	9,043
Total	7	16,962	16,969

**Table 5:** Results of balance tests.

Matching method	Pseudo R^2^	LR χ^2^	Standardized mean differences
Unmatched	0.041	965.71	12.0
Matched			
Nearest-neighbor matching (1:1)	0.000	9.400	0.900
Radius matching	0.000	3.110	0.800
Kernel matching	0.000	5.850	1.1000

**Table 6:** Sensitivity analyses: PSM approach.

Variable	Propensity score matching		
	Nearest neighbor matching	Radius matching	Kernel matching
	(1)	(2)	(3)
Average treatment effect on the treated			
ATT	0.059^***^ (0.016)	0.063^***^ (0.012)	0.065^***^ (0.012)
Matched-sample ordered logistic regression estimates			
WREA	0.139^***^ [0.052, 0.226]	0.156^***^ [0.087, 0.225]	0.157^***^ [0.088,0.226]
Occupation FE	YES	YES	YES
Year FE	YES	YES	YES
Province FE	YES	YES	YES
*N* (treated)	9,036	9,036	9,036
*N* (control)	4,421	7,924	7,926
*N*	13,457	16,960	16,962

Standard errors for ATT are reported in parentheses and computed by psmatch2. For the matched-sample ordered logistic regression, robust standard errors are clustered at the individual level. *N* (Treated) and *N* (Control) denote the numbers of unique treated and control observations retained under each matching specification, respectively. ^***^ indicates significance at the 1% level.

To examine whether the observed association was sensitive to alternative analytical choices, we conducted additional analyses using alternative outcome definitions, an alternative estimation method, and an expanded sample that included agricultural workers. First, we replaced the dependent variable. We used continuous BMI values as a quantitative measure of overweight and obesity status. The results are shown in column (1) of Table 7. We then replaced the Chinese BMI classification criteria with the World Health Organization's BMI classification criteria for Asian populations. The results are presented in column (2) of Table 7. Second, we changed the estimation model and re-estimated the regressions using an ordered Probit model. The results are shown in column (3) of Table 7. Finally, we expanded the sample to include agricultural labor previously excluded from the baseline regression. The results are reported in column (4) of Table 7. Across columns (1) to (4) of Table 7, the direction and statistical significance of the coefficient on WREA were highly consistent with the baseline results.

**Table 7:** Sensitivity analyses: alternative specifications.

Variable	Replacing the dependent variable		Applying an alternative estimation method	Including agricultural labor
	BMI	Obesity_status_2		
	(1)	(2)	(3)	(4)
WREA	0.287^***^ [0.180, 0.395]	0.136^***^ [0.080, 0.191]	0.086^***^ [0.058, 0.115]	0.149^***^ [0.084, 0.215]
Controls	YES	YES	YES	YES
Occupation FE	YES	YES	YES	YES
Year FE	YES	YES	YES	YES
Province FE	YES	YES	YES	YES
N	16,969	16,969	16,969	17,165

Due to space constraints, only coefficient values are reported in the table. ^***^ indicates significance at the 1% level.

### 3.4 Exploratory analyses

#### 3.4.1 Exploratory heterogeneity analyses

Whether the associations of WREA with overweight and obesity differ significantly across population subgroups warrant further investigation. In this section, we conducted exploratory subgroup analyses by sex, age, and educational attainment. A pooled interaction model was employed to examine whether differences across groups were statistically significant.

The gender-stratified regression results are presented in columns (1) and (2) of Table 8. In males, WREA was significantly associated with a higher likelihood of overweight and obesity. The marginal effects were 0.027 (95% CI: 0.016 to 0.038) for overweight and 0.023 (95% CI: 0.014 to 0.032) for obesity. In females, we did not find statistically significant marginal effects of WREA on overweight and obesity. The sample was also divided into three groups according to educational attainment: a low education subgroup (primary school or below), a medium education subgroup (junior or senior high school), and a high education subgroup (college or above). The corresponding subgroup regression results are presented in columns (3) to (5) of Table 8. In the low education subgroup, the estimated marginal effects of WREA were positive on both overweight and obesity but did not reach statistical significance, and their 95% confidence intervals included zero. In contrast, significant positive associations were observed in both the medium and high education subgroups. Moreover, the medium education subgroup (ME for overweight = 0.026, 95% CI: 0.014 to 0.038; ME for obesity = 0.017, 95% CI: 0.009 to 0.026) had larger absolute marginal effects for both overweight and obesity than the high education subgroup (ME for overweight = 0.014, 95% CI: 0.001to 0.027; ME for obesity = 0.009, 95% CI: 0.001 to 0.018). The age-stratified regression results are reported in columns (6) to (8) of Table 8. WREA was significantly associated with a higher risk of overweight and obesity in both the 16–30 and 30–50 age groups. The absolute marginal effects were larger in the 30–50 age group (ME for overweight = 0.028, 95% CI: 0.018 to 0.038; ME for obesity = 0.020, 95% CI: 0.013 to 0.027) than in the 16–30 age group (ME for overweight = 0.018, 95% CI: 0.004 to 0.032; ME for obesity = 0.010, 95% CI: 0.003 to 0.018), indicating a stronger association among middle-aged workers. Among workers aged 50 years and older, the estimated marginal effects of WREA on overweight and obesity were not statistically significant, and their 95% confidence intervals included zero.

**Table 8:** Heterogeneity analysis.

Variable	Gender		Education			Age		
	(1) Male	(2) Female	(3) Primary or below	(4) Junior and senior	(5) College or above	(6) 16–30	(7) 30–50	(8) 50+
Marginal effects of WREA^a^								
Underweight	−0.007^***^ [−0.010, −0.004]	−0.006 [−0.015, 0.001]	−0.003 [−0.008, 0.003]	−0.008^***^ [−0.012, −0.004]	−0.007^**^ [−0.015, −0.000]	−0.018^**^ [−0.031, −0.004]	−0.008^***^ [−0.010, −0.006]	−0.001 [−0.004, 0.001]
Normal weight	−0.043^***^ [−0.060, −0.026]	−0.010 [−0.022, 0.002]	−0.015 [−0.047, 0.017]	−0.035^***^ [−0.052, −0.019]	−0.015^**^ [−0.029, −0.001]	−0.010^**^ [−0.019, −0.002]	−0.040^***^ [−0.054, −0.025]	−0.020 [−0.050, 0.010]
Overweight	0.027^***^ [0.016, 0.038]	0.011 [−0.002, 0.025]	0.010 [−0.012, 0.033]	0.026^***^ [0.014, 0.038]	0.014^**^ [0.001, 0.027]	0.018^**^ [0.004, 0.032]	0.028^***^ [0.018, 0.038]	0.014 [−0.007, 0.034]
Obesity	0.023^***^ [0.014, 0.032]	0.005 [−0.001, 0.011]	0.007 [−0.008, 0.022]	0.017^***^ [0.009, 0.026]	0.009^**^ [0.001, 0.018]	0.010^**^ [0.003, 0.018]	0.020^***^ [0.013, 0.027]	0.008 [−0.004, 0.020]
*P*-value^b^	0.080		0.058			0.037		
Controls	YES	YES	YES	YES	YES	YES	YES	YES
Occupation FE	YES	YES	YES	YES	YES	YES	YES	YES
Year FE	YES	YES	YES	YES	YES	YES	YES	YES
Province FE	YES	YES	YES	YES	YES	YES	YES	YES
*N*	9,946	7,023	2,536	8,890	5,543	3,735	9,670	3,564

^a^Each column reports the marginal effects for different weight statuses in the subgroup regression. ^b^Joint Wald tests assess between-group differences in pooled interaction models. ^**^ and ^***^ indicate significance at the 5% and 1% levels, respectively

#### 3.4.2 Exploratory analyses of the moderating effects

To examine how work and family factors moderate the relationships between WREA and overweight and obesity, this study incorporated interaction terms in the baseline regression model, thereby providing evidence to inform targeted occupational health interventions.

##### 3.4.2.1 Moderating effects of work Factors

Moderating effect of employment status. Workers employed by institutions, those employed by individuals, and self-employed workers differ in their employment arrangements and working conditions, including work schedules, forms of work organization, income stability, and workload variability. On this basis, we incorporated dummy variables for employment by individuals (*casualemp*) and self-employment (*selfemp*), as well as their interaction terms with the exposure variable, into the baseline regression model. The results in column (1) of Table 9 show that the marginal effects of the interaction terms on both overweight and obesity were significantly negative, and their 95% confidence intervals did not include zero. This finding indicates that the positive associations of WREA with overweight and obesity were weaker among self-employed or individually employed workers, suggesting a potential moderating role of employment status.

**Table 9:** Moderating effects of work characteristics and family factors.

Variable	Work factors		Variable	Family factors		
	Employment status	Access to rest-day		Child-rearing burden	Support for parents	
					Economic support	Caregiving support
	(1)	(2)		(3)	(4)	(5)
WREA			WREA			
Underweight	−0.009^***^ [−0.012, −0.005]	−0.008^***^ [−0.011, −0.005]	Underweight	−0.006^***^ [−0.009, −0.003]	−0.006^**^ [−0.012, 0.000]	−0.006^*^ [−0.013, 0.000]
Normal weight	−0.029^***^ [−0.041, −0.017]	−0.026^***^ [−0.036, −0.015]	Normal weight	−0.019^***^ [−0.028, −0.010]	−0.015^**^ [−0.031, 0.000]	−0.015^*^ [−0.032, 0.001]
Overweight	0.023^***^ [0.014, 0.033]	0.021^***^ [0.012, 0.029]	Overweight	0.015^***^ [0.008, 0.022]	0.013^**^ [0.000, 0.026]	0.013^*^ [−0.001, 0.026]
Obesity	0.015^***^ [0.009, 0.021]	0.013^***^ [0.008, 0.019]	Obesity	0.010^***^ [0.005, 0.015]	0.009^**^ [0.000, 0.017]	0.009^*^ [−0.001, 0.018]
WREA^*^casualemp (ref: firmemp)			WREA^*^kid16			
Underweight	0.019^**^ [0.002, 0.035]		Underweight	−0.004^*^ [−0.007, 0.000]		
Normal weight	0.060^**^ [0.006, 0.113]		Normal weight	−0.012^*^ [−0.024, 0.000]		
Overweight	−0.047^**^ [−0.090, −0.005]		Overweight	0.009^*^ [−0.000, 0.019]		
15.6-8.6,-13498.7ptObesity	−0.031^**^ [−0.059, −0.003]		Obesity	0.006^*^ [−0.000, 0.012]		
WREA^*^selfemp (ref: firmemp)			WREA^*^e_support			
Underweight	0.007^**^ [0.002, 0.016]		Underweight		−0.008^***^ [−0.014, −0.004]	
Normal weight	0.023^**^ [0.006, 0.052]		Normal weight		−0.021^***^ [−0.035, −0.010]	
Overweight	−0.018^**^ [−0.042, −0.005]		Overweight		0.018^***^ [0.008, 0.029]	
Obesity	−0.012^**^ [−0.027, −0.003]		Obesity		0.012^***^ [0.005, 0.019]	
WREA^*^restday			WREA^*^c_support			
Underweight		0.009^*^ [−0.001, 0.020]	Underweight			−0.006 [−0.015, 0.004]
Normal weight		0.014^*^ [−0.002, 0.029]	Normal weight			−0.014 [−0.037, 0.010]
Overweight		−0.016^*^ [−0.034, 0.002]	Overweight			0.012 [−0.008, 0.031]
Obesity		−0.007^*^ [−0.015, 0.001]	Obesity			0.008 [−0.006, 0.022]
Controls	YES	YES	Controls	YES	YES	YES
Occupation FE	YES	YES	Occupation FE	YES	YES	YES
Year FE	YES	YES	Year FE	YES	YES	YES
Province FE	YES	YES	Province FE	YES	YES	YES
*N*	16,969	16,965	*N*	16,969	11,080	10,597

Each column reports the marginal effects for different weight statuses. ^*^, ^**^, and ^***^ indicate significance at the 10%, 5%, and 1% levels, respectively.

Moderating effect of access to rest days. We used access to rest days (*restday*) as the moderator, coded 1 for workers who had rest days and 0 for those who did not. This variable and its interaction term with the core independent variable were then included in the baseline regression model. As shown in column (2) of Table 9, the marginal effects of the interaction terms on both overweight and obesity were negative (ME for overweight = −0.016, 95% CI: −0.034 to 0.002; ME for obesity = −0.007, 95% CI: −0.015 to 0.001). However, these estimates were significant only at the 10% level, and both 95% confidence intervals included zero, indicating limited evidence of a moderating effect of access to rest days.

##### 3.4.2.2 Moderating effects of family factors

Moderating effect of child-rearing burden. We constructed a binary variable to measure child-rearing burden, based on whether participants had children younger than 16 (kid16;1 = yes, 0 = no). As shown in Column (3) of Table 9, the estimated marginal associations for the interaction between child-rearing burden and WREA were positive for both overweight and obesity (ME for overweight = 0.009,95% CI: −0.000 to 0.019; ME for obesity = 0.006, 95% CI: −0.000 to 0.019), although the corresponding 95% confidence intervals included zero after rounding. Thus, the results provide only tentative evidence of heterogeneity by child-rearing burden.

Moderating effect of support for parents. Support for parents was assessed across two dimensions: financial support and caregiving support. The financial dimension was defined by whether the respondent provided financial assistance to their parents (*e_support*; 1 = yes, 0 = no). The caregiving dimension was defined by whether the respondent helped their parents with household tasks (*c_support*; 1 = yes, 0 = no). Regression results in column (4) of Table 9 show that the interaction term between financial support and the core independent variable had significant positive marginal effects on both overweight and obesity (ME for overweight = 0.018, 95% CI: 0.008 to 0.029; ME for obesity = 0.012, 95% CI: 0.005 to 0.019). In contrast, the interaction term involving caregiving support was not statistically significant. These findings indicate that the positive associations of WREA with overweight and obesity were stronger among workers with greater financial support responsibilities for their parents, whereas no sufficient evidence was found that caregiving responsibilities for parents could moderate this association.

#### 3.4.3 Exploratory analyses across WREA categories

Given the potential heterogeneity of WREA, we conducted exploratory analyses using two operational classification strategies to examine possible variation in its association with overweight and obesity across WREA categories.

First, we distinguished between types of WREA based on workers' labor contract status, which indicates their potential access to institutional labor protections. Specifically, WREA was classified as contract-based when workers had signed formal labor contracts and as non-contract-based otherwise. Second, we grouped WREA into occupation-related, digitally enabled, and other forms based on their primary drivers. Occupation-related WREA refers to extended availability that arises from the inherent demands of occupations, with primary responsibilities focused on responding to unexpected or urgent situations. Digitally enabled WREA refers to extended availability driven by digital office tools and instant communication technology. Other forms of WREA may arise from organizational norms, competitive pressures, or factors not captured by the first two categories. WREA in occupations that involve emergency services, such as physicians, police officers, and firefighters, was classified as a traditional occupation-related WREA. WREA associated with highly digitalized job was classified as digitally enabled WREA, while the remaining WREA were placed into a distinct category. Job digitalization was measured using three items: the importance of the internet for work (scored on a 1–5 scale, with higher scores indicating greater importance), time spent online via mobile devices, and time spent online via computers. Time spent online via mobile devices and computers was divided into quintiles and assigned scores from 1 to 5, from lowest to highest. We then calculated the arithmetic mean of the three item scores to obtain a composite digitization score. WREA associated with a digitalization score of 3 or higher were defined as digitally enabled WREA, and the rest were classified as other forms. We incorporated dummy variables representing occupation-related, digitally enabled, and other forms of WREA into the baseline regression.

As shown in column (1) of Table 10, both the WREA with and without formal labor contracts were positively associated with overweight and obesity. The marginal effects of the WREA with formal labor contracts (ME for overweight = 0.027, 95% CI: 0.016 to 0.038; ME for obesity = 0.017, 95% CI: 0.010 to 0.024) were numerically larger than the WREA without formal labor contracts (ME for overweight = 0.013, 95% CI: 0.001 to 0.026; ME for obesity = 0.008, 95% CI: 0.001 to 0.016). As reported in column (2) of Table 10, digitally enabled WREA showed a positive association with overweight and obesity at the 1% significance level. Occupation-related WREA were positively associated with overweight and obesity, but the associations were relatively weak and significant at the 10% level. In contrast, no significant association was observed for other forms of WREA.

**Table 10:** Analysis based on different types of WREA.

Variable	(1)	Variable	(2)
Contract-based WREA		Occupation related WREA	
Underweight	−0.011^***^ [−0.016, −0.006]	Underweight	−0.010^*^ [-0.022, 0.002]
Normal weight	−0.033^***^ [−0.046, −0.019]	Normal weight	−0.029^*^ [−0.064, 0.005]
Overweight	0.027^***^ [0.016, 0.038]	Overweight	0.025^*^ [−0.004, 0.054]
Obesity	0.017^***^ [0.010, 0.024]	Obesity	0.015^*^ [−0.003, 0.033]
Non-contract-based WREA		Digitally enabled WREA	
Underweight	−0.005^**^ [−0.010, 0.000]	Underweight	−0.011^***^ [−0.015, −0.006]
Normal weight	−0.016^**^ [−0.031, −0.001]	Normal weight	−0.031^***^ [−0.043, −0.018]
Overweight	0.013^**^ [0.001, 0.026]	Overweight	0.026^**^ [0.015, 0.036]
Obesity	0.008^**^ [0.000, 0.016]	Obesity	0.016^***^ [0.009, 0.022]
		Other WREA	
		Underweight	−0.004 [−0.011, 0.003]
		Normal weight	−0.011 [−0.031, 0.008]
		Overweight	0.009 [−0.007, 0.026]
		Obesity	0.006 [−0.004, 0.016]
Controls	Yes	Controls	Yes
Occupation FE	YES	Occupation FE	YES
Year FE	Yes	Year FE	Yes
Province FE	Yes	Province FE	Yes
*N*	14,262	*N*	13,592

Each column reports the marginal effects for different weight statuses. ^*^, ^**^, and ^***^ indicate significance at the 10%, 5%, and 1% levels, respectively.

#### 3.4.4 Exploratory longitudinal analysis

To further explore the longitudinal association of WREA with subsequent overweight and obesity, we conducted an additional exploratory analysis. Specifically, we regressed the dependent variable from the CFPS2022 dataset on the core independent variable and covariates from the CFPS2020 dataset. We restricted the sample to individuals whose primary work remained unchanged between the two survey waves. The results are reported in Column (1) of Table 11. To account for pre-existing differences in weight status, we additionally adjusted for continuous BMI measured in 2020. The corresponding results are reported in column (2).

**Table 11:** Longitudinal association between WREA and subsequent overweight and obesity.

Variable	(1)	(2)
WREA_2020		
Underweight	−0.005^***^ [−0.009, −0.001]	−0.003 [−0.007, 0.002]
Normal weight	−0.025^***^ [−0.043, −0.007]	−0.014 [−0.033, 0.005]
Overweight	0.017^***^ [0.004,0.029]	0.009 [−0.005, 0.023]
Obesity	0.013^***^ [0.003, 0.023]	0.007 [−0.005, 0.019]
Bmi2020		
Underweight		−0.026^***^ [−0.028, −0.024]
Normal weight		−0.075^***^ [−0.078, −0.073]
Overweight		0.056^***^ [0.054, 0.059]
Obesity		0.045^***^ [0.042, 0.047]
Controls	YES	YES
Occupation FE	YES	YES
Year FE	No	No
Province FE	YES	YES
*N*	4,830	4,796

The dependent variable is the corresponding outcome in 2022, measured in the same way as in the baseline regression. Each column reports the marginal effects for different weight statuses. Standard errors are clustered at the province level. ^***^ indicates significance at the 1% level.

As shown in Table 11, WREA in 2020 was positively associated with overweight and obesity status in 2022 before adjustment for baseline BMI (ME for overweight = 0.017, 95% CI: 0.004 to 0.029; ME for obesity = 0.013, 95% CI: 0.003 to 0.023). However, after further adjustment for baseline BMI, the estimated associations were attenuated and the marginal effects were no longer statistically significant, as their confidence intervals all encompassed zero. (ME for overweight = 0.009, 95% CI: −0.005 to 0.023; ME for obesity = 0.007, 95% CI: −0.005 to 0.019).

## 4 Discussion

### 4.1 Main findings and explanations

We used an ordered logistic regression model to examine the association of WREA with overweight and obesity. The main findings are as follows:

First, WREA was significantly and positively associated with overweight and obesity. These associations remained consistent across a series of sensitivity analyses.

Based on prior literature, we suggest several potential explanations for this association, although these mechanisms were not directly tested in our study due to data limitations. From a psychological perspective, WREA may expose employees to persistent stress and negative emotions (8, 9, 21). Over time, chronic stress and negative emotions could disrupt normal metabolic function and promote fat accumulation (14, 22). Negative emotions may also trigger emotional eating, which could partly explain the positive associations of WREA with overweight and obesity (15). From a behavioral perspective, WREA may disrupt health-related routines. For example, temporary work demands may reduce the time available for activities essential to physical and mental recovery, such as sleep and physical exercise (13). In addition, the uncertainty associated with being constantly on call may intensify negative emotions, thereby impairing sleep quality and increasing emotional eating (12). Such an imbalance between energy intake and energy expenditure may be associated with a greater risk of metabolic health problems. This may partly explain the positive associations of WREA with overweight and obesity.

Second, the associations of WREA with overweight and obesity showed potential variation across worker subgroups. When stratified by sex, WREA was significantly associated with overweight and obesity among men, whereas the marginal effects among women were smaller in magnitude and did not reach statistical significance. This observed pattern may reflect sex differences in awareness of healthy lifestyle practices (23). By educational attainment, workers with a college education or above had lower probabilities of overweight and obesity when exposed to WREA than those with a junior or senior high school education. This discrepancy may be attributable to the fact that more educated workers tend to have greater job autonomy, superior emotional regulation ability, and higher health literacy (24–26). These advantages may attenuate the association of WREA with poorer mental health and less healthy behaviors, which may, in turn, be associated with lower likelihoods of overweight and obesity. Among workers with primary education or below, the findings provide insufficient evidence to establish associations of WREA with overweight or obesity in this subgroup. By age, the associations of WREA with overweight and obesity were stronger among middle-aged workers aged 30–50 than among younger workers aged 16–30. This age-based disparity likely stems from dual work and family burdens faced by middle-aged workers, coupled with a gradual decline in metabolic function (27). Among workers aged 50 years and older, the available evidence was insufficient to establish associations of WREA with overweight and with obesity.

Third, exploratory analyses of the moderating effects suggested that certain work- and family-related factors may moderate the associations of WREA with overweight and obesity. On one hand, the positive association of WREA with overweight and obesity was weaker among workers who were self-employed or employed by individuals. Uncertainty in income and work demands may be a common feature of the working lives of these workers. At the same time, differences in employment arrangements may allow these workers to organize their working hours and work processes more flexibly. Greater familiarity with fluctuating work demands, together with greater flexibility in organizing work, may be associated with fewer disruptions to health-related behaviors and lower psychological stress, potentially explaining the reason for the weaker associations between WREA and overweight and obesity (13). On the other hand, the positive associations of WREA with overweight and obesity were stronger among workers with greater family burdens, particularly those who provide financial support to their parents. One possible explanation concerns time constraint. Workers with heavy family burdens often face more rigid time constraints (28). WREA may exacerbate preexisting work-family role conflict. The combination of WREA and family burdens may be associated with less time for recovery and participation in health promoting activities, which may help explain the observed associations with overweight and with obesity. Another possible explanation concerns psychological stress. The combination of rigid time constraints and financial pressures may be associated with heightened concern over missing work calls or unscheduled job assignments. Chronic psychological stress is usually associated with emotional eating and disruptions to normal metabolic rhythms, which may help explain the stronger associations of WREA with overweight and obesity among workers with greater family burdens (29).

Fourth, exploratory analyses suggested possible variation in the association with overweight and obesity across study-defined WREA categories. When WREA was classified according to workers' labor contract status, contract-based WREA was associated with greater likelihoods of overweight and obesity. One possible explanation is that, to maintain the stability of their employment relationships, workers with formal labor contracts may feel compelled to accommodate requests for availability outside regular working hours and may experience greater pressure. This tendency may be accompanied by less regular daily routines and insufficient recovery, which could be related to the higher likelihoods of overweight and obesity observed in this group. Compared with occupation-related and other forms of WREA, digitally enabled WREA showed a stronger and more statistically significant association with overweight and obesity. One possible explanation is that digital work tools may expand the spatial and temporal boundaries of work, making workers more readily available for unexpected work tasks at any time and location (30). The widespread adoption of digital communication tools may further accelerate the transmission of work demands, leaving workers with limited time to respond and complete tasks. Collectively, these factors may be associated with greater work-life conflict and persistent psychological exhaustion, which may help explain the stronger associations of digitally enabled WREA with overweight and obesity (31). In contrast, traditional occupation-related WREA may, in some cases, reflect occupational choice and may involve more structured scheduling arrangements and greater predictability. These characteristics may be associated with lower levels of chronic stress and fewer disruptions to workers' daily routines.

Fifth, we did not find sufficient evidence of a longitudinal association between WREA and subsequent overweight or obesity after adjustment for baseline BMI. The associations between WREA and subsequent overweight or obesity were attenuated, and the confidence intervals included zero after adjustment for baseline BMI. This pattern suggests that the observed associations in longitudinal analysis without baseline BMI adjustment may be partly explained by pre-existing differences in body weight.

### 4.2 Comparison with other studies

Previous research has extensively examined the associations of various nonstandard work schedules with overweight and with obesity, including overtime, telework, and flexible work schedules (32–35). Although these nonstandard work schedules take various forms, the overall evidence points to a consistent story: increased after-hours availability and greater work encroachment on personal life are positively associated with a higher likelihood of obesity. Li et al. (33) examined the association between long working hours and obesity through a meta-analysis. Their analysis revealed that reduced non-working time was associated with shorter sleep duration and reduced physical activity, hindering employees' recovery from occupational fatigue (33). These time constraints may also adversely affect health-related behaviors, such as alcohol consumption and smoking, thereby further increasing the risk of obesity. Almandoz et al. (34) further reported that partial telework which requires frequent transitions between home and the workplace may disrupt daily routines (34). Such disruption may increase exposure to multiple risk factors associated with weight gain, including stress, depressive symptoms, greater consumption of takeout or comfort foods, and overeating

Our study shifted the analytical focus from specific nonstandard work arrangements to their shared underlying condition: work-related extended availability. We found that WREA was positively associated with overweight and with obesity, a finding consistent with prior research on overtime, telework, and flexible work schedules. Importantly, by capturing the common essence of these varied practices, our findings provide a more integrated perspective on the associations between modern work arrangements and overweight and obesity.

In addition, findings linking WREA to poorer sleep quality, emotional exhaustion, and state of recovery also indicate that WREA may be associated with higher risks of overweight and of obesity (11, 12, 36, 37). These studies establish a crucial theoretical framework for our investigation. Building on this foundation, we extend this line of inquiry to metabolic health by examining the potential associations of WREA with overweight and obesity. Additionally, we examine how this relationship may differ across population groups and work and life contexts.

### 4.3 Limitations

This study expands the scope of occupational obesity research by focusing on WREA. However, several limitations exist in this study. First, our analysis relied on pooled cross-sectional data from two survey waves. Therefore, the study results primarily reflect correlations among the research variables rather than strict causal effects. Second, more detailed exposure measures, such as on-call frequency, response duration, location, and work intensity, were not available due to the survey data. This limitation prevents a comprehensive and accurate quantification of WREA. Third, BMI relied on self-reported anthropometrics may bias the observed associations of WREA with overweight and with obesity. Future research should employ objective measurements to validate these findings. Fourth, measurement error and omitted variable bias remain possible. Due to data limitations, we used several proxy variables in this study, such as dietary variables. And some relevant confounders were unavailable, so residual confounding cannot be fully ruled out. Although multiple covariates were controlled, the instrument's exclusion restriction cannot be formally verified, which remains a limitation of our IV analysis. Lastly, our exploratory and indirect classification of WREA may cause exposure misclassification, highlighting the need for validated, direct measures in future research.

## 5 Conclusion

This study clarifies the association of work-related extended availability (WREA) with overweight and with obesity, highlighting an emerging occupational health risk. The findings indicate that WREA is associated with higher likelihoods of overweight and obesity. This association may be more pronounced among men, individuals with a junior or senior high school education, and those aged 30–50 years. Exploratory interaction analyses suggested that the association of WREA with overweight and obesity may vary according to employment status and the financially support for parents. WREA with a formal labor contract, as well as digitally enabled WREA, is associated with higher likelihoods of overweight and obesity and therefore warrants particular attention in occupational health governance. Additionally, we did not find sufficient evidence of a longitudinal association between WREA and subsequent overweight or obesity.

These findings suggest that WREA may warrant greater attention in occupational health research and practice, particularly as this work practice becomes increasingly common. Governments could consider strengthening the monitoring and regulation of such emerging forms of labor practice and continuously evaluating their potential association with workers' overall wellbeing. At the same time, employers should be encouraged to explore more humane management strategies to help employees achieve a healthier balance between work and personal life.

## Data Availability

Publicly available datasets were analyzed in this study. This data can be found here: https://cfpsdata.pku.edu.cn/#/home.
