# Supplementary material for: Associations of work-related extended availability with overweight and obesity among Chinese workers
Source: Front Public Health. 2026 Sep 3;14:1907128. doi: 10.3389/fpubh.2026.1907128 (PMC13582586; doi:10.3389/fpubh.2026.1907128)
Supplement: Supplementary file 1 [file Supplementary_File_1.docx]

Supplementary Material

# 1  Covariate balance before and after propensity score matching

We report the covariate balance test results and Love plots for all three matching methods in Supplementary Tables 1–3 and Supplementary Figures 1–3, respectively. After applying 1:1 nearest-neighbor matching, radius matching, and kernel matching, the absolute standardized mean differences (SMD) for all covariates fell below the conventional 10% threshold. Specifically, under 1:1 nearest-neighbor matching, the maximum absolute SMD decreased from 28.3% to 2.2%, and the mean absolute SMD dropped from 12.0% to 0.9%. Radius matching yielded similar improvements, reducing the maximum absolute SMD to 2.2% and the mean absolute SMD to 0.8%. Kernel matching reduced the maximum absolute SMD to 2.5% and the mean absolute SMD to 1.1%.Overall, the matching quality was satisfactory across all three methods.

**Supplementary Table 1. covariate balance before and after 1:1 nearest-neighbor matching**

| **Covariate** | **Sample** | **Treated mean** | **Control mean** | **SMD (%)** |
| --- | --- | --- | --- | --- |
| Age | U | 40.252 | 37.468 | 25.6 |
|  | M | 40.238 | 40.362 | -1.1 |
| Age2 | U | 1740.5 | 1521.1 | 24.6 |
|  | M | 1739.1 | 1751.6 | -1.4 |
| Gender | U | 0.65078 | 0.51236 | 28.3 |
|  | M | 0.65051 | 0.64498 | 1.1 |
| Education | U | 11.089 | 11.262 | -4.3 |
|  | M | 11.089 | 11.075 | 0.4 |
| Spouse | U | 0.81477 | 0.75360 | 14.9 |
|  | M | 0.81463 | 0.81507 | -0.1 |
| Residence | U | 0.64857 | 0.65077 | -0.5 |
|  | M | 0.64885 | 0.64885 | 0.0 |
| Occupation | U | 3.9456 | 4.2236 | -15.1 |
|  | M | 3.9479 | 3.9384 | 0.5 |
| Province | U | 37.864 | 36.744 | 7.5 |
|  | M | 37.851 | 37.985 | -0.9 |
| Year | U | 2021 | 2021 | -4.5 |
|  | M | 2021 | 2020.9 | 1.6 |
| Income | U | 10.280 | 10.317 | -4.5 |
|  | M | 10.281 | 10.266 | 1.7 |
| Social_ Status | U | 2.9193 | 2.7904 | 13.6 |
|  | M | 2.9181 | 2.8969 | 2.2 |
| Income_Status | U | 2.8970 | 2.9209 | -1.0 |
|  | M | 2.8970 | 2.8997 | -0.1 |
| Mean absolute SMD (%) | **U** |  |  | **12.0** |
|  | **M** |  |  | **0.9** |
| Maximum absolute SMD (%) | **U** |  |  | **28.3** |
|  | **M** |  |  | **2.2** |

Notes: U = unmatched sample; M = matched sample; SMD = standardized mean difference. Following 1:1 nearest-neighbor matching, the absolute SMD was below 10% for every covariate. The maximum absolute SMD fell from 28.3% to 2.2%, and the mean absolute SMD fell from 12.0% to 0.9%.

**Supplementary Table 2. Covariate balance before and after radius matching**

| **Covariate** | **Sample** | **Treated mean** | **Control mean** | **SMD (%)** |
| --- | --- | --- | --- | --- |
| Age | U | 40.252 | 37.468 | 25.6 |
|  | M | 40.238 | 40.102 | 1.3 |
| Age2 | U | 1740.5 | 1521.1 | 24.6 |
|  | M | 1739.1 | 1727.8 | 1.3 |
| Gender | U | 0.65078 | 0.51236 | 28.3 |
|  | M | 0.65051 | 0.64739 | 0.6 |
| Education | U | 11.089 | 11.262 | -4.3 |
|  | M | 11.089 | 11.118 | -0.7 |
| Spouse | U | 0.81477 | 0.75360 | 14.9 |
|  | M | 0.81463 | 0.81173 | 0.7 |
| Residence | U | 0.64857 | 0.65077 | -0.5 |
|  | M | 0.64885 | 0.65036 | -0.3 |
| Occupation | U | 3.9456 | 4.2236 | -15.1 |
|  | M | 3.9479 | 3.9395 | 0.5 |
| Province | U | 37.864 | 36.744 | 7.5 |
|  | M | 37.851 | 37.808 | 0.3 |
| Year | U | 2021 | 2021 | -4.5 |
|  | M | 2021 | 2021 | 0.7 |
| Income | U | 10.280 | 10.317 | -4.5 |
|  | M | 10.281 | 10.283 | -0.3 |
| Social_ Status | U | 2.9193 | 2.7904 | 13.6 |
|  | M | 2.9181 | 2.8974 | 2.2 |
| Income_Status | U | 2.8970 | 2.9209 | -1.0 |
|  | M | 2.8970 | 2.8806 | 0.7 |
| Mean absolute SMD (%) | **U** |  |  | **12.0** |
|  | **M** |  |  | **0.8** |
| Maximum absolute SMD (%) | **U** |  |  | **28.3** |
|  | **M** |  |  | **2.2** |

Notes: U = unmatched sample; M = matched sample; SMD = standardized mean difference. The absolute SMD was below 10% for every covariate after radius matching. The maximum absolute SMD fell from 28.3% before matching to 2.2% after matching. The mean absolute SMD fell from 12.0% to 0.8%.

**Supplementary Table 3. Covariate balance before and after kernel matching**

| **Covariate** | **Sample** | **Treated mean** | **Control mean** | **SMD (%)** |
| --- | --- | --- | --- | --- |
| Age | U | 40.252 | 37.468 | 25.6 |
|  | M | 40.238 | 40.017 | 2.0 |
| Age2 | U | 1740.5 | 1521.1 | 24.6 |
|  | M | 1739.1 | 1720.4 | 2.1 |
| Gender | U | 0.65078 | 0.51236 | 28.3 |
|  | M | 0.65051 | 0.64463 | 1.2 |
| Education | U | 11.089 | 11.262 | -4.3 |
|  | M | 11.089 | 11.121 | -0.8 |
| Spouse | U | 0.81477 | 0.75360 | 14.9 |
|  | M | 0.81463 | 0.81067 | 1.0 |
| Residence | U | 0.64857 | 0.65077 | -0.5 |
|  | M | 0.64885 | 0.65087 | -0.4 |
| Occupation | U | 3.9456 | 4.2236 | -15.1 |
|  | M | 3.9479 | 3.9580 | -0.5 |
| Province | U | 37.864 | 36.744 | 7.5 |
|  | M | 37.851 | 37.765 | 0.6 |
| Year | U | 2021 | 2021 | -4.5 |
|  | M | 2021 | 2021 | 0.4 |
| Income | U | 10.280 | 10.317 | -4.5 |
|  | M | 10.281 | 10.286 | -0.6 |
| Social_ Status | U | 2.9193 | 2.7904 | 13.6 |
|  | M | 2.9181 | 2.8944 | 2.5 |
| Income_Status | U | 2.8970 | 2.9209 | -1.0 |
|  | M | 2.8970 | 2.8783 | 0.8 |
| Mean absolute SMD (%) | **U** |  |  | **12.0** |
|  | **M** |  |  | **1.1** |
| Maximum absolute SMD (%) | **U** |  |  | **28.3** |
|  | **M** |  |  | **2.5** |

Notes: U = unmatched sample; M = matched sample; SMD = standardized mean difference. Following kernel matching, the absolute SMD was below 10% for every covariate. The maximum absolute SMD fell from 28.3% to 2.5%, and the mean absolute SMD fell from 12.0% to 1.1%.

**Supplementary Figure 1. Love Plot for 1:1 Nearest-Neighbor Matching**

**Supplementary Figure 2. Love Plot for Radius Matching**

**Supplementary Figure 3. Love Plot for Kernel Matching**
